# APE-Gen: A Fast Method for Generating Ensembles of Bound Peptide-MHC Conformations

**DOI:** 10.3390/molecules24050881

**Published:** 2019-03-02

**Authors:** Jayvee R. Abella, Dinler A. Antunes, Cecilia Clementi, Lydia E. Kavraki

**Affiliations:** 1 Department of Computer Science, Rice University, Houston, TX 77005, USA; j.abella@rice.edu (J.R.A.); dinler@rice.edu (D.A.A.); 2 Center for Theoretical Biological Physics, Rice University, Houston, TX 77005, USA; cecilia@rice.edu; 3 Department of Chemistry, Rice University, Houston, TX 77005, USA

**Keywords:** MHC class I, ensemble, molecular docking, peptide binding

## Abstract

The Class I Major Histocompatibility Complex (MHC) is a central protein in immunology as it binds to intracellular peptides and displays them at the cell surface for recognition by T-cells. The structural analysis of bound peptide-MHC complexes (pMHCs) holds the promise of interpretable and general binding prediction (i.e., testing whether a given peptide binds to a given MHC). However, structural analysis is limited in part by the difficulty in modelling pMHCs given the size and flexibility of the peptides that can be presented by MHCs. This article describes APE-Gen (Anchored Peptide-MHC Ensemble Generator), a fast method for generating ensembles of bound pMHC conformations. APE-Gen generates an ensemble of bound conformations by iterated rounds of (i) anchoring the ends of a given peptide near known pockets in the binding site of the MHC, (ii) sampling peptide backbone conformations with loop modelling, and then (iii) performing energy minimization to fix steric clashes, accumulating conformations at each round. APE-Gen takes only minutes on a standard desktop to generate tens of bound conformations, and we show the ability of APE-Gen to sample conformations found in X-ray crystallography even when only sequence information is used as input. APE-Gen has the potential to be useful for its scalability (i.e., modelling thousands of pMHCs or even non-canonical longer peptides) and for its use as a flexible search tool. We demonstrate an example for studying cross-reactivity.

## 1. Introduction

The Class I Major Histocompatibility Complex (MHC) is a protein that plays a central role in our adaptive immune system [1]. MHCs bind to intracellular peptides, about 8–11 amino acids in length, and the combined peptide-MHC (pMHC) complex is transported to the cell surface. Surveilling T-cells then inspect the pMHCs to determine whether a given cell is diseased or healthy. Diseased cells will tend to display a set of peptides that are different from the types of peptides that are presented by healthy cells, and an immune response is triggered if a T-cell is able to recognize such a differing peptide. Studying pMHCs has potential applications for immunotherapy, which leverages this mechanism to deliver precise treatments against certain diseases, such as cancer [2].

One direction in studying pMHCs is binding prediction, since not every peptide binds to a given MHC. There are thousands of different MHC allotypes found in the human population, each with its own preference for the kinds of peptides that will bind, which is in turn determined by the MHC sequence. Experimental methods alone cannot cover the sheer number of combinations of pMHCs possible (i.e., all possible peptides presented by all MHC allotypes in the population), making computational methods an attractive complementary approach. The leading approaches for computational binding prediction are based on using sequence as the basis for prediction, typically through the use of neural networks, and are trained using a dataset of experimentally-determined binding affinities [3,4,5]. While sequence-based methods allow for rapid prediction of pMHC binding, their performance for allotypes not included in training sets is difficult to quantify [6].

An alternative computational approach is based on analyzing the structure of pMHCs. Structure-based methods have the potential to be more interpretable and general across pMHCs, since binding predictions are based on the existence of structural features, such as atomic interactions. The structural analysis can be based on structures derived from X-ray crystallography experiments for example, and there are about 600 pMHC crystal structures available in the Protein Data Bank (PDB) at the time of this writing. However, given that the number of pMHC crystal structures only covers a small fraction of pMHC combinations, computational methods have been developed to model conformations of peptides bound to MHCs [7].

Toward this end, molecular docking tools can be used to generate bound pMHC structures [8]. Molecular docking aims to predict the most likely conformation a given ligand (e.g., peptide) will take in the binding site of a receptor (e.g., MHC). The main challenge that molecular docking methods face with pMHCs is handling the high-dimensional conformational space of peptides in a computationally efficient manner. Please note that the receptor conformation must also be considered, as the sidechains in the binding site of the MHC can rearrange depending on the peptide conformation. Popular molecular docking software, such as AutoDock Vina [9], do search in conformational space with genetic algorithms, and candidate conformations (peptide plus MHC) are evaluated with the help of a scoring function [10]. The output is a single conformation or a select few conformations that are considered high quality by the scoring function. The accuracy of such methods is then assessed by comparing the returned conformations with those found in the reference crystal structure, typically with a metric known as root mean square deviation (RMSD) that computes a distance between two conformations. In the context of pMHCs, general molecular docking methods [11] as well as methods built specifically for pMHCs have been applied to model bound pMHC conformations. Examples of docking methods built specifically for pMHCs include using *a priori* knowledge of bound pMHC conformations to limit the conformational search [12,13,14] or incorporating a pMHC-specific scoring function [15]. For a more comprehensive discussion of molecular docking for pMHCs or more generally how structure-based methods have been applied to pMHCs, we refer the interested reader to a recently published review [7].

However, a largely ignored component in the structural analyses is that biomolecules such as pMHCs are not static in solution. The pMHC system may adopt multiple conformations, and thus subsequent analyses involving only a single conformation per pMHC could lead to misleading conclusions. In [16], the authors used a technique known as ensemble refinement to generate alternative conformations of pMHCs that are still consistent with the X-ray crystallography experiment. They found that when structural analyses are instead done with conformations produced from ensemble refinement, alternative conclusions can be formed due to the existence of different interactions between peptide and MHC.

Therefore, in this work, we are interested in developing a method that can generate an ensemble of conformations, as opposed to simply producing the most probable one as done with docking-based methods. Structural analysis of pMHCs can then be done on the ensemble, which takes into account the previously neglected flexibility of the peptide within the MHC binding site. Having access to such an ensemble could allow one to explore alternative bound conformations, which the pMHC may adopt naturally in solution or in response to interacting T-cells. Currently there is a lack of computationally efficient methods that can produce such an ensemble of plausible (clash-free) pMHC conformations. A naive way of generating an ensemble would be to rerun docking tools to generate multiple bound pMHC conformations. However, molecular docking methods simply were not built to perform this task since they are relatively slow to rerun often given the size and flexibility of peptide ligands, and do not aim to produce diverse bound conformations. Additional work would need to be done with molecular docking tools to keep track of what conformations have already been produced at a particular point. Another method that could be used is molecular dynamics, which simulate the interactions between atoms through time [17,18,19]. However, besides the fact that this method requires a bound pMHC conformation to begin with, molecular dynamics is computationally demanding in that it requires massive amounts of computational resources to explore physiologically relevant timescales [20].

To develop a method that is both computationally efficient and can produce diverse bound pMHC conformations, we gained insight from two previously noted observations. The first takes advantage of the fact that the ends of the peptide are known to be anchored at particular pockets within the MHC binding site. Therefore, if the ends of the peptide are more or less in fixed positions, the majority of the conformational search can focus on finding conformations for the middle of the peptide. This insight turns the problem into a loop modelling problem, for which there are methods already developed [21,22,23,24], and indeed this insight has also been used by other methods for modelling pMHCs [12,13,14]. A method that focuses on only the middle portions of the peptide makes it more efficient as it limits the conformational search. However, loop modelling software typically works by fixing the surrounding conformation, meaning that the peptide conformations are generated with a fixed receptor conformation. Thus, the peptide conformations that are sampled by loop modelling are biased by the receptor conformation. The second observation allows our method to overcome this bias. In [25], DOCKTOPE overcomes docking with a rigid receptor conformation by alternating docking with energy minimization. Since loop modelling is done with respect to a given fixed conformation, our method can similarly alternate loop modelling with energy minimization. Multiple rounds of loop modelling followed by energy minimization then ensures a more diverse sampling of peptide conformations, since a different receptor conformation can be used in each round.

These insights allowed us to develop APE-Gen (Anchored Peptide-MHC Ensemble Generator), a fast method for generating bound pMHC conformations. APE-Gen generates an ensemble of bound conformations by iterated rounds of loop modelling followed by energy minimization, and only requires the sequence of the peptide and MHC as input. A single round consists of i) anchoring the ends of a given peptide near known pockets in the binding site of the MHC, ii) sampling peptide backbone conformations with loop modelling, and then iii) performing energy minimization to fix steric clashes. The energy minimization is done with a scoring function typically used for docking [26] that models electrostatic, hydrogen bonding, solvation, and hydrophobic effects. The energy minimization is done over the peptide conformation as well as the receptor sidechains in the binding site. At the end of a round, the sampled conformations are pooled together with the conformations sampled from previous rounds, and the conformation with the lowest energy is used as input to the next round. The combination of loop modelling followed by energy minimization allows APE-Gen to generate a diverse ensemble of bound pMHC conformations that can be used for further structural analysis. APE-Gen is fast and naturally takes into account receptor flexibility through the energy minimization. We validate APE-Gen by assessing its ability to sample the conformation found in the corresponding crystal structures, even when only sequence information is used as input. We also discuss a few application scenarios that showcase the scalability and flexibility of APE-Gen. APE-Gen is open-source and freely available at https://github.com/KavrakiLab/APE-Gen.

## 2. Results

### 2.1. Reproducing Crystal Structures

**Figure 1:**
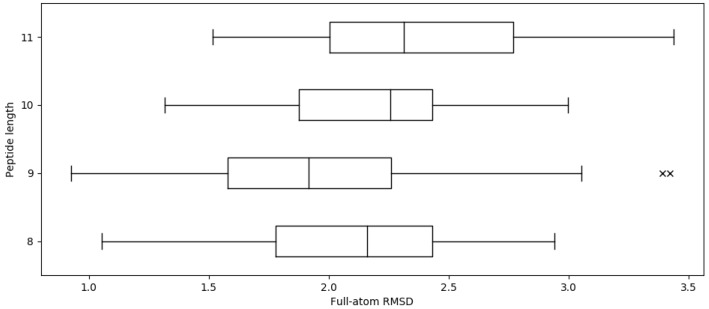
Distribution of final full-atom RMSD across various peptide lengths. The average full-atom RMSD across whole dataset is 2.02 Å. There are a total of 93, 317, 91, and 34 structures of 8-mers, 9-mers, 10-mers, and 11-mers, respectively.

We tested APE-Gen on its ability to find the native conformation as determined with X-ray crystallography. The pMHC crystal structures available in the PDB were determined with the help of the IMGT/3Dstructure database [27] and a total of 603 entries were found. We excluded structures that had missing domains, gaps in sequence, modified residues, or structures with peptides longer than 11 amino acids, leaving a total of 535 pMHCs. APE-Gen was run for each of these pMHCs, using the sequence of the peptide along with the receptor conformation found in the crystal structure. Please note that while the correct receptor conformation is used as input (resembling redocking experiments in molecular docking), APE-Gen samples different receptor sidechains within every round through the energy minimization step. APE-Gen was run for 10 rounds, and we report the RMSD of the sampled peptide conformation that is closest to the one found in the crystal structure (Figure 1).

For a given peptide and MHC, APE-Gen takes approximately 5 min per round to generate tens of conformations on 12 processor cores at 2.83 GHz. The average full-atom RMSD across whole dataset is 2.02 ± 0.46 Å (Cα RMSD 0.91 ± 0.32 Å). Given that the resolution of X-ray crystallography experiments is around 2 Å, these results show that APE-Gen can sample conformations that are found in crystal structures. Figure 1 shows the distributions of the full-atom RMSD values across different peptide lengths. While there are some pMHCs for which APE-Gen was only able to sample conformations around 3 Å or greater, the low reported Cα RMSD values show that most of the differences are likely concentrated in the sidechains of the peptide. Small errors in sidechain configuration can be easily corrected in post-processing steps, and most pipelines for structural analysis include a short minimization with a more accurate energy function. From Figure 1, we notice that the average RMSD values are larger for 10-mers and 11-mers compared to 9-mers. This makes intuitive sense since longer peptides have more degrees of freedom, and thus, lower the likelihood that APE-Gen can sample a conformation near the crystal structure.

### 2.2. Using Only Sequence Information

Next we tested the ability of APE-Gen to sample a crystal-like structure when only using sequence information (i.e., no structural data on the MHC receptor). Two MHCs were tested and input conformations were obtained using homology modelling: HLA-A*02:01 was modelled with a structure of HLA-A*24:02 (PDB code 3I6L), and HLA-A*24:02 was modelled with a structure of HLA-A*02:01 (PDB code 1DUZ), using the software MODELLER [28]. Then, we assessed the ability of APE-Gen to sample the crystal conformation for pMHCs of HLA-A*24:02 or HLA-A*02:01 that have crystal structures. These two allotypes were chosen because they are the most prevalent in the human population, and HLA-A*02:01 has the greatest number of crystal structures available in the PDB. APE-Gen was run for 10 rounds, and we measure the RMSD of the conformation that is most similar to the one found in the crystal structure. The mean full-atom RMSD across the 13 pMHCs for HLA-A*24:02 was 2.20 ± 0.21 Å (Cα RMSD 1.10 ± 0.17 Å), and across 123 pMHCs for HLA-A*02:01 was 2.18 ± 0.34 Å (Cα RMSD 1.12 ± 0.29 Å). We also compared the difference in full-atom RMSD between using the modelled MHC versus the actual crystal structure. For HLA-A*02:01, the average difference was 0.35 Å, while the average difference HLA-A*24:02 was a mere 0.05 Å. In some cases, APE-Gen was even able to sample a conformation closer to the crystal when using the modelled MHC. Therefore, we see that while the average RMSDs are slightly worse than when using an actual crystal structure of the receptor, the average RMSDs are still at acceptable values.

### 2.3. Application: Modelling Thousands of pMHCs

One potential application of APE-Gen takes advantage of its ability to model thousands of pMHCs. As mentioned in the Introduction, there is considerable interest in being able to predict whether a given peptide will bind to a particular MHC, or even predicting the strength of peptide binding. We wanted to test the limits of applicability and see if APE-Gen as it is currently built would be able to do large-scale screening of peptides (i.e., to discriminate between so-called binders and non-binders). Each conformation produced goes through a scoring function [10,26], and so APE-Gen can score a particular peptide using the best scoring conformation that was sampled. We wanted to test whether the scoring function used within APE-Gen (SMINA scoring function [26]) could be used to classify binders and non-binders, where known binders would produce better scores than known non-binders. APE-Gen was used to model 11234 HLA-A*02:01-restricted peptides for which there are known experimental binding affinities [4]. The receptor conformation found in PDB code 1DUZ was used as the input HLA-A*02:01 conformation. To save on computing time, APE-Gen was run for a single round per peptide. Running APE-Gen for a single round opens the possibility of insufficient sampling, but the average relative difference between a binder and a nonbinder is assumed to stay the same since the final score used can only be better with more sampling. APE-Gen required almost 24 h of wall clock time on a computing cluster across 45 nodes with 12 processing cores each to produce a score for each peptide. Unfortunately, there is only a weak correlation between the scores obtained using APE-Gen and the true binding affinities (Spearman R: 0.255). In other words, there was no clear separation between binders and non-binders using the scores. However, this experiment serves as a proof-of-concept on the ability of APE-Gen to perform large-scale modeling of pMHC complexes, regardless of available structural data. APE-Gen could be combined with pMHC-specific scoring functions or used to generate training data sets for machine learning based methods to conduct structure-based binding affinity prediction.

### 2.4. Application: Modelling a 15-mer Peptide

The speed of APE-Gen allows one to model many different peptides within a reasonable time. In addition to this scalability, we wanted to test the ability of APE-Gen to model longer, non-canonical peptides (>11 amino acids). Longer peptides have more degrees of freedom, in the form of rotatable bonds, which presents a computational challenge for molecular docking tools as the conformational space is higher dimensional and it becomes more difficult to identify high quality conformations with respect to a scoring function. For APE-Gen in particular, the likelihood of sampling a given structure decreases since the loop modelling and energy minimization steps are less likely to sample the correct backbone and sidechain conformations, respectively. As a proof-of-concept, we wanted to see if APE-Gen could sample the crystal structure given enough time.

**Figure 2:**
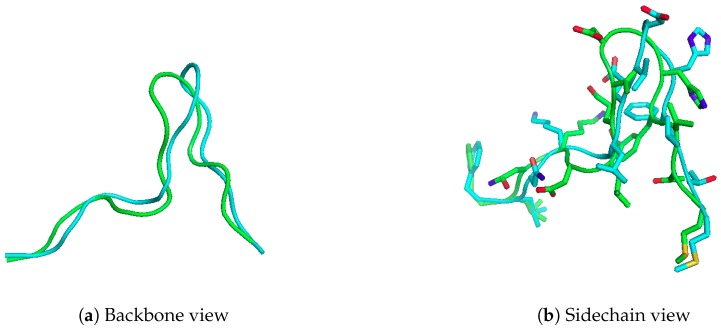
Modelling 15-mer peptide, FLNKDLEVDGHFVTM, onto HLA-A*02:01. The crystal structure is in blue (PDB code 4U6Y), while the modelled structure is in green. APE-Gen can generate a model of the peptide to 2.82 Å full-atom RMSD (1.57 Å Cα RMSD)

Here we present the results of running APE-Gen on the 15-mer “FLNKDLEVDGHFVTM”, which is known to bind to HLA-A*02:01, and checking whether APE-Gen can sample a conformation similar to the one reported in PDB (PDB code 4U6Y). This 15-mer is a naturally processed peptide that has also been shown to be recognized by T-cells [29]. Therefore, the ability to model this peptide and other longer, non-canonical peptides can lead to further insights into T-cell immunity. In Figure 2, we show the conformation sampled that was closest to the conformation found in the crystal structure after a total of 20 rounds. The anchor template used was taken from a different structure (PDB code 4U6X), and the receptor conformation was taken from PDB code 1DUZ. APE-Gen was able to produce a model of the peptide to 2.82 Å full-atom RMSD (1.57 Å Cα RMSD). The total time taken by APE-Gen was about 200 min on an Intel Core i7-4790. Please note that APE-Gen produced not only this structure, but an ensemble of conformations that could better capture the intrinsic flexibility of such long peptide ligands. Knowledge of these alternative possible conformations could be key to understand T-cell responses against non-canonical peptide binders.

### 2.5. Application: Studying Cross-Reactivity

**Figure 3:**
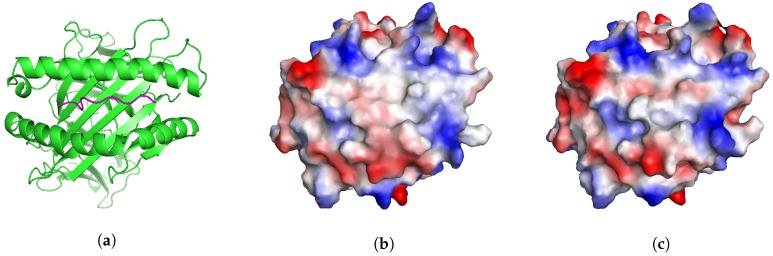
View of T-cell interacting interface. (**a**) Ribbon view of MAGEA3 (pink) bound to HLA-A*01:01 (green), PDB code 5BRZ (**b**) Electrostatic surface of MAGEA3 bound to HLA-A*01:01, PDB code 5BRZ (**c**) Electrostatic surface of Titin-derived self-peptide bound to HLA-A*01:01 (APE-Gen model). APE-Gen can search for conformations Titin-derived self-peptide that produce similar pMHC interfaces to the reference MAGEA3 conformation. Electrostatic surfaces are computed using PyMOL’s “Protein Contact Potential” feature.

We present a different application that illustrates another potential use of APE-Gen. MAGEA3 is a well-known melanoma-associated antigen that binds to HLA-A*01:01 [30]. This particular pMHC was originally intended as a target for T-cell-based cancer immunotherapy. Unfortunately, in some cases, the T-cells targeting MAGEA3 were also able to recognize and target an unrelated Titin-derived self-peptide, which has resulted in the death of at least 4 patients in recent clinical trials [31,32]. Similar off-target reactions have been found in other studies [33,34]. This phenomenon where two different pMHCs are recognized by the same T-cell is known as cross-reactivity [35]. Two pMHCs are more likely to be cross-reactive if their T-cell interacting interfaces (Figure 3a) are “similar” [36]. One way to assess the similarity of two T-cell interacting interfaces is by inspecting their electrostatic surfaces [36]. Therefore, if the structure of a potential pMHC target is known, we could search for other potentially dangerous cross-reactive pMHCs by inspecting their electrostatic surfaces [37].

We show that APE-Gen could, in principle, be a tool that can search for conformations of a pMHC that present similar T-cell interacting interfaces to a reference one. In this scenario, the structure of MAGE-A3 bound to HLA-A*01:01 is known (Figure 3a, PDB code 5BRZ) and we can compute the electrostatic surface of its T-cell interacting interface (Figure 3b). We run APE-Gen on the self-peptide derived from Titin, bound to HLA-A*01:01, with the goal of sampling a conformation that presents a similar surface to the reference (MAGEA3). APE-Gen was run until the backbone conformation of the self-peptide matched the backbone conformation of the MAGEA3 peptide, as measured when the Cα RMSD between the two peptides falls below 1 Å. Only one round of APE-Gen was needed and the minimum backbone RMSD conformation was used to generate electrostatic surfaces for analysis (Figure 3c). Electrostatic surfaces are computed by mapping electrostatic potentials to the solvent-accessible surface of the T-cell interacting interface. Figure 3 shows that the two systems present qualitatively similar electrostatic surfaces, which is consistent with the fact that the two peptides are known to be cross-reactive [30]. Given its scalability, we anticipate that APE-Gen could be used in the future to conduct structure-based screenings for potentially dangerous cross-reactive targets, as part of the development of new T-cell-based immunotherapies.

## 3. Materials and Methods

**Figure 4:**
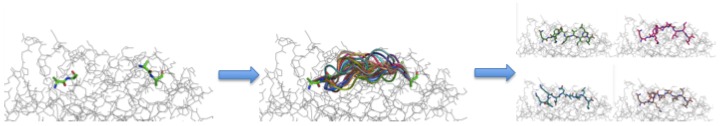
Steps within a single round of APE-Gen. From left to right: anchor alignment, peptide backbone sampling, and energy minimization. After a round is complete, the highest quality conformation as determined by the scoring function is used to initialize the next round.

As mentioned in the Introduction, APE-Gen consists of iterated rounds of three steps: (i) anchoring the ends of a given peptide near known pockets in the binding site of the MHC, (ii) sampling peptide backbone conformations with loop modelling, and then (iii) performing energy minimization to fix steric clashes. For detailed information, APE-Gen is open-source and freely available at https://github.com/KavrakiLab/APE-Gen. In this section, we describe the steps that APE-Gen takes to generate an ensemble of bound conformations for a particular pMHC. The steps for a given round can be summarized in Figure 4. First, we describe how the input is prepared, followed by a description of the steps, and then how APE-Gen transitions between rounds.

### 3.1. Input Preparation

Ultimately, all the method requires as input to generate bound pMHC complexes is the sequence of the peptide ligand and the sequence of the MHC receptor. If there is a known conformation of the MHC from the PDB, this structure can be used as input and it would have a positive impact on the results. If not, APE-Gen will use MODELLER and a template to generate a model for the MHC of interest [28]. Given a template MHC structure and the sequence of the MHC receptor, MODELLER searches for conformations of the MHC receptor that are favorable according to MODELLER’s internal DOPE score [28] and multiple conformations are generated. More precisely, only conformations of the alpha chain are modelled, since the sequence of the β2-microglobulin chain is the same across allotypes. We then use the best scoring conformation according to the DOPE score as input to the first round. Please note that the generated MHC conformations are typically similar to the template MHC structure. Thus, the template MHC structure used largely affects the results of the homology modelling. The ideal template for a given allotype to choose would belong to an allotype of the same supertype. MHC allotypes belonging to the same supertype are known to have similarities in the kinds of peptides that bind to them [38]. Otherwise the structure of the MHC receptor obtained by other means (such as crystallography) can be used as input.

### 3.2. Anchor Alignment

As mentioned in the Introduction, the ends of a bound peptide are usually “anchored” to the MHC at particular pockets in the binding site. With the sequence of the peptide ligand and the structure of the MHC receptor, APE-Gen places terminal atoms of the peptide backbone onto the receptor using a template pMHC conformation. The structure of the MHC receptor is aligned to the template pMHC, and the coordinates of the terminal atoms of the peptide backbone found in the template are transferred onto the MHC receptor after alignment. Please note that the pMHC template here is used to determine the coordinates of the peptide anchors, while the template mentioned in the previous section is used to generate a model of the MHC structure. APE-Gen uses the terminal atoms of the first and last two residues of the peptide found in the template, since two residues at each terminus are also the minimum number required for the subsequent loop modelling step. The template pMHC used depends on the length of the peptide sequence, where APE-Gen uses a template with a bound peptide of the same length as the input sequence. This is done since longer peptides bind with an extended “bulge” along the middle of the conformation, which affects the position of the termini backbone atoms, especially for the inner terminal atoms. The template for a given n-mer was chosen by finding the n-mer pMHC structure from PDB that has the minimum average distance to every other n-mer pMHC structure from PDB. The PDB codes for the templates are 2VAA, 1DUZ, 1I4F, and 2NW3 for 8-mers, 9-mers, 10-mers, and 11-mers, respectively. Custom templates are also possible, including the ability to use the highest quality structure sampled from the previous rounds. Higher n-mers can be modelled with APE-Gen, up to 15-mers; however higher n-mers are more susceptible to alternative binding poses where the peptide termini are in atypical locations on the MHC receptor [39].

### 3.3. Peptide Backbone Sampling

In this step, various backbone conformations of the peptide are generated using loop modelling software. Any loop modelling software could be used in this step, but in this work we use a recently developed implementation known as RCD [24]. The underlying algorithm of RCD is based on Random Coordinate Descent, a modification of the more popular CCD or Cyclic Coordinate Descent [22]. The RCD algorithm achieves fast conformational sampling by introducing more randomization into the optimization, tries to close the loop from both ends, and has a fast loop conformation update method with spinor matrices and geometric filters [24]. The software that implements this algorithm is freely available and used by APE-Gen without modification. In this work 100 peptide backbone conformations are sampled per round with a RMSD tolerance of 1.0 Å (i.e., the residues in contact with the middle of the backbone are allowed to move at most 1.0 Å).

### 3.4. Sidechain Sampling and Energy Minimization

With the backbone conformations obtained from the previous step, sidechains are added to the peptide using PDBFixer [40]. PDBFixer internally attempts to add sidechains in a manner that avoids steric clashes. Then, with full-atom conformations of the pMHC, APE-Gen performs energy minimization of the pMHC using SMINA [26]. SMINA is a molecular docking tool that is included in the APE-Gen’s pipeline because of its fast “local search” protocol. SMINA refines the conformation of the peptide and the sidechains of the MHC by performing energy minimization using its internal scoring function. The scoring function used by SMINA resembles a simplified forcefield with empirical terms and models electrostatic, hydrogen bonding, solvation, and hydrophobic effects [26]. Every conformation from the previous step is run through PDBFixer followed by SMINA’s local search feature to produce a candidate bound pMHC conformation. This step is how APE-Gen incorporates receptor conformational changes in response to different peptide conformations. Please note that some pMHC conformations get filtered out in this step as SMINA cannot fix clashes (e.g., clashes introduced by RCD or PDBFixer). Note also that the energy minimization using SMINA has no constraint on the positions of the anchors and can end up producing significantly different peptide conformations (greater than 2 Å change). Thus, conformations that do not feature the peptide anchors in the correct pockets of the MHC are filtered out as a post-processing step.

### 3.5. Running APE-Gen for Multiple Rounds

A single round of running APE-Gen consists of performing the previously described steps of anchor alignment, backbone sampling, and energy minimization. Note again that while receptor flexibility is taken into account in the energy minimization step, the receptor is kept rigid during the step prior when sampling peptide backbone conformations. As mentioned in the Introduction, different peptide backbone conformations may be sampled in the loop modelling step given a different receptor conformation since sidechains in the receptor may be blocking off regions in the binding site for the peptide. Therefore, one way to be more exhaustive in sampling peptide backbone conformations is to input a different receptor conformation in subsequent rounds of running APE-Gen. One convenient way to obtain a different conformation is to use the best scoring pMHC conformation sampled at the end of a given round. Thus, the receptor conformation to be used as input is taken from the best scored pMHC conformation sampled from the previous round. This creates diversity in the conformations sampled across rounds, since different receptor conformations are used for each subsequent loop modelling step. Another way to create diversity is to input a different anchor template for the peptide. This can be done in a similar way for the input receptor conformation, where the best scoring pMHC conformation sampled in the previous round is used to obtain the anchor template for the next round. This feature may be useful if one has reason to believe that a given peptide binds in a different manner (i.e., binding to different pockets in the MHC), which may be more common for higher n-mers or particular MHC allotypes [39]. For the results presented in this work, we have kept this feature disabled. The complete ensemble that APE-Gen computes is the combination of all the bound conformations generated at each round.

## 4. Discussion and Conclusions

APE-Gen stands out as a method that can quickly generate bound conformations of pMHCs given only sequence information. As alluded to in the Results section, APE-Gen has a wide array of potential applications. As a tool, APE-Gen can rapidly sample native-like conformations. The scalability of APE-Gen allows the modelling of thousands of different pMHCs within a reasonable timeframe. Additionally, non-canonical longer peptides (up to 15-mers) can also be modelled by APE-Gen, which is an extremely difficult task for traditional molecular docking approaches due to the additional degrees of freedom. The modelled ensemble of bound conformations can then be used as datasets for further structural analyses.

First, we evaluated APE-Gen on its ability to sample a conformation that is similar to a reference crystal structure. Current general-purpose molecular docking tools, such as AutoDock Vina [9], simply cannot reach the scalability of APE-Gen without invoking some domain-specific knowledge, and so the same task cannot be performed with it. As shown in the Results, APE-Gen can sample a conformation that is similar to the one found in the crystal structure across all the pMHC crystal structures available in the PDB. As a point for comparison, the performance of APE-Gen is comparable to GradDock, a docking tool developed specifically for pMHCs that also features a pMHC-specific scoring function [15], in its ability to generate conformations that have low full-atom RMSDs to a reference crystal structure. While our results show that the produced ensembles include conformations similar to those found in crystal structures, selecting such conformations out of this ensemble is a non-trivial task. For instance, the highest quality conformations in the ensembles produced by APE-Gen (using the SMINA scoring function) are not necessarily the same conformations that are nearest to the ones found in the corresponding crystal structure. This is not a problem specific to APE-Gen, and the top scoring conformations derived from other methods may also be dissimilar to the reference crystal conformation [41]. Future work could investigate the use of other scoring functions, particularly pMHC-specific functions that may be able to better align the quality of a conformation with the crystal-like ones.

However, as mentioned in the Introduction, the structural analyses of pMHCs using a single conformation can be misleading. Interactions between peptide and MHC may be missed when only considering a single conformation and entropic effects are ignored in general. APE-Gen is a step toward the structural analyses of pMHCs in an ensemble fashion. However, the generated ensemble is by no means an optimal one. In fact, the conformations contained within can be viewed as rather coarse, given the nature of the sampling process and the use of the SMINA scoring function. As a result, it is difficult to assign a “weight” to each conformation.

Nevertheless, APE-Gen now provides a rapid way to generate an ensemble of plausible pMHC conformations and enables new kinds of analysis. For instance, in Section 2.3, we investigated the use of the scoring function as a possible way to classify binders from non-binders, where the predicted binding affinity of a particular pMHC is taken as the best score of the highest quality conformation from the ensemble. While our results show that APE-Gen was unable to reliably do binding prediction, the ensembles produced by APE-Gen may lead to future work in improving structure-based binding prediction methods. The models produced by APE-Gen can be used as training sets for future scoring functions that aim to classify binders from non-binders or even predict binding affinities. Future work could also focus on how the ensemble as a whole could be used in predicting binding affinities, as the use of the ensemble could be a way to include previously neglected effects of peptide flexibility.

We have also shown a use case of APE-Gen as a conformational search tool. In the context of cross-reactivity, we have shown how APE-Gen can be used in principle to search for conformations that produce similar “looking” interfaces to a reference pMHC. Other analyses could be possible as one could simply filter through the conformations generated by APE-Gen to fit within some pipeline or run the APE-Gen method until some desirable conformation has been found. The search aspect of APE-Gen could be improved in future work by either making sampling more directed or incorporating some notion of memory to prevent re-sampling similar conformations.

Finally, another exciting application of APE-Gen is the ability to initialize molecular dynamics simulations from multiple diverse starting conformations as it only requires sequence information to produce models. The results of a given simulation may be heavily biased by the starting conformation, and it is becoming more apparent that molecular dynamics simulations should be instead run in an ensemble fashion [42]. A new class of methods known as adaptive sampling are gaining popularity, where many short parallel simulations are iteratively restarted in a principled way to achieve some goal [20,43,44,45]. APE-Gen has the potential to be an ideal companion for adaptive sampling methods that will enable the study of any pMHC system with molecular dynamics.

## Abbreviations

MHC	Major Histocompatibility Complex
pMHC	peptide-MHC
PDB	Protein data bank
RMSD	Root mean square deviation
Cα	alpha-carbon

## References

[1] Rock K.L., Reits E., Neefjes J. (2016). Present Yourself! By MHC Class I and MHC Class II Molecules. Trends Immunol..

[2] Hu Z., Ott P.A., Wu C.J. (2018). Towards personalized, tumour-specific, therapeutic vaccines for cancer. Nat. Rev. Immunol..

[3] Nielsen M., Lundegaard C., Worning P., Lauemøller S.L., Lamberth K., Buus S., Brunak S., Lund O. (2003). Reliable prediction of T-cell epitopes using neural networks with novel sequence representations. Protein Sci..

[4] O’Donnell T.J., Rubinsteyn A., Bonsack M., Riemer A.B., Laserson U., Hammerbacher J. (2018). MHCflurry: Open-Source Class I MHC Binding Affinity Prediction. Cell Syst..

[5] Andreatta M., Nielsen M. (2018). Bioinformatics tools for the prediction of T-cell epitopes. Methods Mol. Biol..

[6] Luo H., Ye H., Ng H.W., Shi L., Tong W., Mendrick D.L., Hong H. (2015). Machine Learning Methods for Predicting HLA-Peptide Binding Activity. Bioinform. Biol. Insights.

[7] Antunes D.A., Abella J.R., Devaurs D., Rigo M.M., Kavraki L.E. (2018). Structure-based methods for binding mode and binding affinity prediction for peptide-MHC complexes. Curr. Top. Med. Chem..

[8] Antunes D.A., Devaurs D., Kavraki L.E. (2015). Understanding the challenges of protein flexibility in drug design. Expert Opin. Drug Discov..

[9] Trott O., Olson A.J. (2010). AutoDock Vina: Improving the speed and accuracy of docking with a new scoring function, efficient optimization, and multithreading. J. Comput. Chem..

[10] Liu J., Wang R. (2015). Classification of current scoring functions. J. Chem. Inf. Model..

[11] Antunes D.A., Moll M., Devaurs D., Jackson K.R., Lizee G., Kavraki L.E. (2017). DINC 2.0: A New Protein-Peptide Docking Webserver Using an Incremental Approach. Cancer Res..

[12] Rosenfeld R., Zheng Q., Vajda S., DeLisi C. (1993). Computing the Structure of Bound Peptides: Application to Antigen Recognition by Class I Major Histocompatibility Complex Receptors. J. Mol. Biol..

[13] Rognan D., Lauemøller S.L., Holm A., Buus S., Tschinke V. (1999). Predicting Binding Affinities of Protein Ligands from Three-Dimensional Models: Application to Peptide Binding to Class I Major Histocompatibility Proteins. J. Med. Chem..

[14] Tong J.C., Tan T.W., Ranganathan S. (2004). Modeling the structure of bound peptide ligands to major histocompatibility complex. Protein Sci..

[15] Kyeong H.H., Choi Y., Kim H.S. (2018). GradDock: rapid simulation and tailored ranking functions for peptide-MHC Class I docking. Bioinformatics.

[16] Fodor J., Riley B.T., Borg N.A., Buckle A.M. (2018). Previously Hidden Dynamics at the TCR-Peptide-MHC Interface Revealed. J. Immunol..

[17] Knapp B., Demharter S., Esmaielbeiki R., Deane C.M. (2015). Current status and future challenges in T-cell receptor/peptide/MHC molecular dynamics simulations. Brief. Bioinform..

[18] Bello M., Campos-Rodriguez R., Rojas-Hernandez S., Contis-Montes de Oca A., Correa-Basurto J. (2015). Predicting peptide vaccine candidates against H1N1 influenza virus through theoretical approaches. Immunol. Res..

[19] Bello M., Correa-Basurto J. (2016). Energetic and flexibility properties captured by long molecular dynamics simulations of a membrane-embedded pMHCII-TCR complex. Mol. Biosyst..

[20] Paul F., Wehmeyer C., Abualrous E.T., Wu H., Crabtree M.D., Schoneberg J., Clarke J., Freund C., Weikl T.R., Noe F. (2017). Protein-peptide association kinetics beyond the seconds timescale from atomistic simulations. Nat. Commun..

[21] Shehu A., Kavraki L.E., Clementi C. (2007). On the characterization of protein native state ensembles. Biophys. J..

[22] Canutescu A.A., Dunbrack R.L. (2003). Cyclic coordinate descent: A robotics algorithm for protein loop closure. Protein Sci..

[23] Shehu A., Kavraki L.E. (2012). Modeling Structures and Motions of Loops in Protein Molecules. Entropy.

[24] Chys P., Chacon P. (2013). Random Coordinate Descent with Spinor-matrices and Geometric Filters for Efficient Loop Closure. J. Chem. Theory Comput..

[25] Rigo M.M., Antunes D.A., Vaz de Freitas M., Fabiano de Almeida Mendes M., Meira L., Sinigaglia M., Vieira G.F. (2015). DockTope: A Web-based tool for automated pMHC-I modelling. Sci. Rep..

[26] Koes D.R., Baumgartner M.P., Camacho C.J. (2013). Lessons learned in empirical scoring with smina from the CSAR 2011 benchmarking exercise. J. Chem. Inf. Model..

[27] Ehrenmann F., Kaas Q., Lefranc M.P. (2010). IMGT/3Dstructure-DB and IMGT/DomainGapAlign: A database and a tool for immunoglobulins or antibodies, T cell receptors, MHC, IgSF and MhcSF. Nucleic Acids Res..

[28] Webb B., Sali A. (2017). Protein Structure Modeling with MODELLER. Methods Mol. Biol..

[29] Hassan C., Chabrol E., Jahn L., Kester M.G., de Ru A.H., Drijfhout J.W., Rossjohn J., Falkenburg J.H., Heemskerk M.H., Gras S. (2015). Naturally processed non-canonical HLA-A*02:01 presented peptides. J. Biol. Chem..

[30] Raman M.C., Rizkallah P.J., Simmons R., Donnellan Z., Dukes J., Bossi G., Le Provost G.S., Todorov P., Baston E., Hickman E. (2016). Direct molecular mimicry enables off-target cardiovascular toxicity by an enhanced affinity TCR designed for cancer immunotherapy. Sci. Rep..

[31] Linette G.P., Stadtmauer E.A., Maus M.V., Rapoport A.P., Levine B.L., Emery L., Litzky L., Bagg A., Carreno B.M., Cimino P.J. (2013). Cardiovascular toxicity and titin cross-reactivity of affinity-enhanced T cells in myeloma and melanoma. Blood.

[32] Cameron B.J., Gerry A.B., Dukes J., Harper J.V., Kannan V., Bianchi F.C., Grand F., Brewer J.E., Gupta M., Plesa G. (2013). Identification of a Titin-derived HLA-A1-presented peptide as a cross-reactive target for engineered MAGE A3-directed T cells. Sci. Transl. Med..

[33] Morgan R.A., Chinnasamy N., Abate-Daga D., Gros A., Robbins P.F., Zheng Z., Dudley M.E., Feldman S.A., Yang J.C., Sherry R.M. (2013). Cancer regression and neurological toxicity following anti-MAGE-A3 TCR gene therapy. J. Immunother..

[34] van den Berg J.H., Gomez-Eerland R., van de Wiel B., Hulshoff L., van den Broek D., Bins A., Tan H.L., Harper J.V., Hassan N.J., Jakobsen B.K. (2015). Case report of a fatal serious adverse event upon administration of T cells transduced with a MART-1-specific T-cell receptor. Mol. Ther..

[35] Degauque N., Brouard S., Soulillou J.P. (2016). Cross-reactivity of TCR repertoire: Current concepts, challenges, and implication for allotransplantation. Front. Immunol..

[36] Antunes D.A., Rigo M.M., Freitas M.V., Mendes M.F.A., Sinigaglia M., Lizee G., Kavraki L.E., Selin L.K., Cornberg M., Vieira G.F. (2017). Interpreting T-Cell Cross-reactivity through Structure: Implications for TCR-Based Cancer Immunotherapy. Front Immunol..

[37] Antunes D.A., Rigo M.M., Silva J.P., Cibulski S.P., Sinigaglia M., Chies J.A., Vieira G.F. (2011). Structural in silico analysis of cross-genotype-reactivity among naturally occurring HCV NS3-1073-variants in the context of HLA-A*02:01 allele. Mol. Immunol..

[38] Mumtaz S., Nabney I.T., Flower D.R. (2017). Scrutinizing human MHC polymorphism: Supertype analysis using Poisson-Boltzmann electrostatics and clustering. J. Mol. Graph. Model..

[39] Guillaume P., Picaud S., Baumgaertner P., Montandon N., Schmidt J., Speiser D.E., Coukos G., Bassani-Sternberg M., Filippakopoulos P., Gfeller D. (2018). The C-terminal extension landscape of naturally presented HLA-I ligands. Proc. Natl. Acad. Sci. USA.

[40] Eastman P., Swails J., Chodera J.D., McGibbon R.T., Zhao Y., Beauchamp K.A., Wang L.P., Simmonett A.C., Harrigan M.P., Stern C.D. (2017). OpenMM 7: Rapid development of high performance algorithms for molecular dynamics. PLoS Comput. Biol..

[41] Ramirez D., Caballero J. (2018). Is It Reliable to Take the Molecular Docking Top Scoring Position as the Best Solution without Considering Available Structural Data?. Molecules.

[42] Bowman G.R., Ensign D.L., Pande V.S. (2010). Enhanced modeling via network theory: Adaptive sampling of Markov state models. J. Chem. Theory Comput..

[43] Doerr S., De Fabritiis G. (2014). On-the-fly learning and sampling of ligand binding by high-throughput molecular simulations. J. Chem. Theory Comput..

[44] Preto J., Clementi C. (2014). Fast recovery of free energy landscapes via diffusion-map-directed molecular dynamics. Phys. Chem. Chem. Phys..

[45] Hruska E., Abella J.R., Nuske F., Kavraki L.E., Clementi C. (2018). Quantitative comparison of adaptive sampling methods for protein dynamics. J. Chem. Phys..

